# Arterio-ureteral fistula: a nationwide cross-sectional questionnaire analysis

**DOI:** 10.1007/s00345-021-03910-3

**Published:** 2022-01-22

**Authors:** Tycho M. T. W. Lock, Kyara Kamphorst, Roderick C. N. van den Bergh, Frans L. Moll, Jean-Paul P. M. de Vries, Rob T. H. Lo, Gérard A. P. de Kort, Rutger C. G. Bruijnen, Pieter Dik, Simon Horenblas, Laetitia M. O. de Kort

**Affiliations:** 1 Department of Urology, University Medical Centre Utrecht, P.O. BOX 85500, 3508 GA Utrecht, The Netherlands; 2 Department of Urology, St. Antonius Hospital, Koekoekslaan 1, 3435 CM Nieuwegein, The Netherlands; 3 Division of Vascular Surgery, Department of Surgery, University Medical Centre Utrecht, P.O. BOX 85500, 3508 GA Utrecht, The Netherlands; 4 Division of Vascular Surgery, Department of Surgery, University Medical Centre Groningen, Hanzeplein 1, 9713 GZ Groningen, The Netherlands; 5 Department of Interventional Radiology, University Medical Centre Utrecht, P.O. BOX 85500, 3508 GA Utrecht, The Netherlands; 6 Department of Pediatric Urology, Wilhelmina Children’s Hospital UMC Utrecht, Utrecht, The Netherlands; 7 Division of Pediatric Urology and Andrology, Department of Pediatric Surgery, I.M. Sechenov First Moscow State Medical University, Moscow, Russia; 8 Department of Urology, The Netherlands Cancer Institute, Plesmanlaan 121, 1066 CX Amsterdam, The Netherlands

**Keywords:** Hematuria, Stents, Arterio-ureteral fistula, Endovascular procedures, Incidence

## Abstract

**Purpose:**

Arterio-ureteral fistula (AUF) is an uncommon diagnosis, but potentially lethal. Although the number of reports has increased over the past two decades, the true incidence and contemporary urologists’ experience and approach in clinical practice remains unknown. This research is conducted to provide insight in the incidence of AUF in The Netherlands, and the applied diagnostic tests and therapeutic approaches in modern practice.

**Methods:**

A nationwide cross-sectional questionnaire analysis was performed by sending a survey to all registered Dutch urologists. Data collection included information on experience with patients with AUF; and their medical history, diagnostics, treatment, and follow-up, and were captured in a standardized template by two independent reviewers. Descriptive statistics were used.

**Results:**

Response rate was 62% and 56 AUFs in 53 patients were reported between 2003 and 2018. The estimated incidence of AUF in The Netherlands in this time period is 3.5 AUFs per year. Hematuria was observed in all patients; 9% intermittent microhematuria, and 91% presenting with, or building up to massive hematuria. For the final diagnosis, angiography was the most efficient modality, confirming diagnosis in 58%. Treatment comprised predominantly endovascular intervention.

**Conclusion:**

The diagnosis AUF should be considered in patients with persistent intermittent or massive hematuria.

**Supplementary Information:**

The online version contains supplementary material available at 10.1007/s00345-021-03910-3.

## Introduction

Arterio-ureteral fistula (AUF) is a rare but potentially lethal complication, where a direct connection between artery or vascular graft and ureter exists. First clinical presentation is often intermittent hematuria, without any additional symptoms, which frequently ceases without any treatment [1, 2]. AUF may also present with massive hematuria, especially during endoscopic ureteral instrumentation and/or stent replacement.

Different risk factors for AUF have previously been described [1]: a combination of arterial and ureteral stenting, arterial pseudo-aneurysm, previous urinoma formation, previous radiation, chronically infected tissue, and abdominal (vascular) surgery. This may lead to friction due to arterial pulsations against a ureter with an indwelling stent of frail and less flexible tissue.

Neither the European Association of Urology (EAU) nor the American Urological Association (AUA) include recommendations on AUF diagnosis or treatment.

We observed an increase in the incidence of AUF in our personal practice, which was confirmed by the reports in medical literature.

The aim of the current study is to estimate true incidence in The Netherlands, contemporarily applied diagnostic, and therapeutical approaches.

A cross-sectional questionnaire approach was applied, in which a questionnaire was sent to all registered Dutch urologists. We focused on the incidence in The Netherlands, and the contemporary diagnostic modalities and treatment options. Based on the results of the questionnaire and in the absence of guidelines, recommendations and algorithm for clinical urologic practice will be presented.

## Materials and methods

### Patients

A digital questionnaire was sent to all (*n* = 398) registered Dutch urologists in December 2018 via the Dutch Urology Association (NVU). A reminder was sent out in February 2019. Questions included years in practice, and the encounter with patients suggestive of AUF in the period 2003–2018 (Supplementary Fig. 1).

Anonymized details of patients were retrospectively collected via a standard item list (Supplementary Fig. 2) by two independent reviewers (TL and KK). Details included patient details, and data on diagnostics and treatment.

### Definitions

AUF was defined as the confirmed presence of an abnormal passageway between a ureter and any artery or a previously inserted vascular graft. Only cases of confirmed cases of AUF were included. Excluded from analysis were cases describing fistulas between the ureter and any venous structure, fistulas between the bladder and an artery, cases in which ureteral damage was observed but no true fistula, and fistulas between the intestinal part of an ileal conduit urinary diversion and an artery.

End points were death or loss to follow-up. Death was defined as AUF specific (due to massive hemorrhage or infection directly post-diagnosis) or death due to other causes.

### Ethics

The Ethics Committee of the University Medical Center Utrecht approved this study (reference number WAG/mb/19/042283). The Dutch Urological Association approved questionnaire distribution among urologists. The need for individual consent was waived.

### Statistical analysis

Descriptive statistics were used (SPSS version 27.0).

## Results

### Questionnaire

A total of 247 urologists of the 398 registered urologists in The Netherlands completed the questionnaire, amounting to a response rate of 62%. The years of practice in this group ranged from 1 to 30 years, with a mean of 17.1 years.

In total 56 AUFs were reported in 53 patients. The 6 AUFs in 6 patients diagnosed between 2003 and 2007 we presented earlier [2] were included in the incidence calculation, but not further described in this article. Of 14 cases, the exact details could not be retrieved. The details of the remaining 36 AUFs in 33 patients are presented in Tables 1 and 2.

**Table 1:** demographics, diagnostics and AUF location

No./age/gender	Oncologic history, surgery, IC	Gy	Vascular surgery history	IUS (mo)	Hematuria	Accompanying symptoms	AUF neg	AUF pos	AUF location	(PS)A
1/84/F			R CIA TEA		Mass		0	UCS, X-RPG	R U–R CIA	
2/52/F			L AIB AFB, L sten EIA	6	Interm		0	MRI	L U–L EIA	
3/85/F			AFB		Mass	Clotret	1	CTA	R U–R EIA	PSA
4/69/M	Bladder, CP, yes	60			Mass		0	CTA	R U–R EIA	PSA
5/63/M	Bladder, CP, yes			30	Mass	Clotret	0	Angio	L U–L CIA	
6/62/M			AFB		Interm-Mass		0	CTA	R U–R CIA	A
7/52/F			AIB	24	Mass		3	PET-CT/CTA	R U–R CIA	
8/76/M			AIB	48	Interm-Mass	Clotret, sepsis	1	OK	R U–R CIA	
9/74/M			R CIA desobstr, R KS	13	Interm-Mass		1	Angio	R U–R CIA	PSA
10/69/M	Bladder, CP, yes		R EIA defect, repair		Interm-Mass	Shock	1	OK	R U–R EIA	PSA
11/76/M	Prostate, P + AE, yes	72		30	Interm-Mass	Shock	3	Angio	L U–R CIA	
12/38/F	Cervical, H, no	76		15	Interm-Mass	Clotret	1	Angio	R U–R EIA	
13/71/F	Ovarian, H, no	68		38	Interm-Mass		2	Angio	I R U–R CIA, II L U–L CIA	
14/62/M			R CIA stent, AIB	36	Mass	Melena	1	OK	R U–R CIA–Colon	PSA
15/58/F			AIB	36	Interm-Mass	Clotret, flank pain	3	Angio	R U–R CIA	PSA
16/83/M	Bladder, CP, yes			36	Interm-Mass		3	I:Angio, II:CTA	I L U–L CIA, II L U–L IIA	
17/68/F				15	Interm	Urosepsis	0	CTA	R U–R CIA	
18/63/M	Prostate, P + C, yes			24	Interm-Mass		0	Angio	L U–L CIA	
19/77/F	Cervical, AE, yes	68		18	Mass		2	I, II: Angio	I L U–Aorta, II IC–R EIA	
20/68/M	Prostate, P, no	78		14	Mass		3	Angio	L U–L CIA	
21/80/F	Cervical, AE, yes	64		6	Mass		0	Angio	R U–R IIA	
22/72/M	Anal, APR, no	64		24	Interm-Mass		3	Angio	L U–L CIA	
23/47/F	Cervical, AE, yes	55		44	Mass		3	Angio	L U–L CIA	
24/72/F			R IFB	41	Interm		0	CTA	R U–R CIA	
25/69/M	Bladder, CP, yes		EVAR	30	Mass		0	Angio	R U–R CIA	
26/46/F	Cervical, H + AE, yes	72		54	Mass		0	OK	L U–L EIA	
27/58/M				18	Interm-Mass	Clotret	0	Angio	L U–L CIA	
28/72/M	Bladder, CP, yes			62	Mass		2	Angio	L U–L CIA	
29/54/F	Cervical, H + AE, yes	50		36	Interm-Mass		0	Angio	L U–L CIA	
30/73/M			LS	72	Interm-Mass	Clotret, flank pain	0	Postmortem	L U–L CIA	
31/75/M	Rectal, LAR, no	25		48	Interm-Mass		5	Clinic	I L U–L IIA, II R U–R CIA	
32/70/M	Bladder, CP, yes		L bypass	14	Mass		0	Angio	L U–L CIA	PSA
33/64/F	Rectal + Anal, LAR, no	65		47	Interm-Mass	Flank pain	2	I:X-APG/Angio, II: CTA	I L U–L CIA, II R U–R CIA	PSA

*AE* anterior exenteration, *AFB* aortofemoral bypass, *AIB* aortoiliac bypass, *Angio* angiography, *APR* abdominoperineal resection, *C rad.* cystectomy, *CIA* common iliac artery, *Clinic* no radiographic confirmation, *Clotret* clot retention, *CP rad.* cystoprostatectomy, *CTA* computed tomography angiography, *Desobstr* desobstruction, *EIA* external iliac artery, *EVAR* endovascular aneurysm repair, *Gy* radiation in gray, *H rad.* hysterectomy, *IC* ileal conduit, *IFB* iliofemoral bypass, *IIA* internal iliac artery, *Interm* intermittent, *Interm-Mass* first intermittent, later massive, *IUS* indwelling ureteral stent, *KS* kissing stent, *L* left, *LAR* low anterior resection, *LS* lumbar sympathectomy, *Mass* massive, *Mo* months, *OK* operatively, *P* prostatectomy, *(PS)A* (pseudo)aneurysm involvement, *R* right, *Sten* stenosis, *TEA* thrombendarteriectomy, *U* ureter

**Table 2:** treatment, outcome, follow-up (FU)

*N*	Unnec	Open treatment	Endovascular treatment	Emb IIA	Uro	FU	FU time	Reason death	Post-OK
1	Nephr		WALLGRAFT™ 10 × 50 + 12 × 50			Surv	48 m		
2		Rem./IFB (GSV)			UU	Surv	54 m		
3		Femfem			UU	Died	4 m	Sepsis	
4		Dacron 8 × 50 mm				Died	4 m	Sepsis	
5			WALLGRAFT™ 12 × 50 + Zenith^®^ 14 × 80			Died	84 m	Car accident	
6			VIABAHN^®^ 13 × 100			Surv	21 m		
7			Advanta V12^®^ 10 × 60	Yes		Surv	2 m		
8		Lig. R CIA, Femfem				Died	35 m	Cardiovasc	Afunctional kidney
9		Rem./Nitinol 10 × 79, Advanta 10 × 59 (KS)			U-lysis	Surv	28 m		E Coli sepsis
10			Endo 9 × 38	Yes		Died	11 m	Cancer (bladder M+)	
11			Jaguar™ 45 × 12			AUF	6 d	Post-OK: pulm insuf	
12			VIABAHN^®^ 60 × 10	Yes		Died	3 m	Sepsis	IL FBV AE death
13			I VIABAHN^®^, II VIABAHN^®^ 80 × 10	Yes		Surv	50 m		8 m: second AUF
14		Rem./Dacron 12 × 50 + VP				Surv	36 m		
15			FLUENCY^®^ 12 × 60			Died	34 m	Cancer (lung)	
16			I FLUENCY^®^ 14 × 40, II Endo 12 × 40			Surv	15 m		12 m: second AUF
17			Endo 9 × 40			Died	1 m	Pneumatosis intest	
18			FLUENCY^®^ 12 × 60			Died	50 m	Cardiovasc	
19		I VP, omental wrap	II FLUENCY^®^ 10 × 60	Yes		Surv	36 m		6 m: ACF
20			VIABAHN^®^ 12 × 60		Balloon	Surv	72 m		
21		Lig. R IIA	Advanta V12^®^ 8 × 59		URI	Died	2 m	Sepsis, cardiovasc	
22			VIABAHN^®^ 8 × 50			Surv	60 m		
23			FLUENCY^®^ 12 × 60			Surv	60 m		
24		Rem./IFB (GSV)				Surv	59 m		
25			VIABAHN^®^	Yes		Died	1 m	Sepsis, cardiovasc	
26		IFB			URI	Surv	61 m		
27	Nephr		EVAR limb			Surv	72 m		
28			FLUENCY^®^ 10 × 50	Yes		Surv	60 m		
29			VIABAHN^®^ 10 × 50	Yes		Surv	18 m		
30						AUF	1.5 d	Mass hemorrhage	
31			II GORE^®^ EXCLUDER^®^15 × 70	I Yes		Surv	92 m		< 1 d: second AUF
32			BeGraft^®^ 10 × 38			Died	41 m	Possible COVID	30 m: infection graft
33			I FLUENCY^®^ 10 × 60, II FLUENCY^®^ 8 × 40	Yes	Allium	Died	32 m	Cancer (Anal M +)	Rec.: 2 extra endo stents

*ACF* arterial-conduit fistula, *AE* anterior exenteration, *AUF* AUF-related death, *Balloon* temporary balloon tamponade, *cardiovasc* cardiovascular, *CIA* common iliac artery, *d* day, *emb* embolization, *endo* endovascular stent (name not known), *FBV* fistula bladder–vagina, *femfem* femoral–femoral bypass, *GSV* great saphenous vein, *IFB* iliofemoral bypass, *IL* intestinal leak, *intest* intestinalis, *KS* kissing stent, *lig* ligation, *m* months, *mass* massive, *n* case number, *nephr* nephrectomy, *pulm insuff* pulmonary insufficiency, *rec* recurrent hematuria, *rem./* removal of old stents, *surv* survived, *u-lysi*s ureterolysis, *unnec* unnecessary treatment, *URI* ureteral reimplantation, *UU* ureteroureterostomy, *VP* venous patch, *x* diameter × length (mm) stent

Thus, within a timeframe of 16 years 56 AUFs were identified. On average 3.5 recognized AUFs/year. The Netherlands had 16.2 million inhabitants in 2003 and 17.5 million in 2021 [3].

### Demographics, diagnostics and AUF location (*Table **1*)

The male to female ratio was 55%:45%. A history of oncological treatment and pelvic radiation was observed in 61 and 39%, respectively, with a mean radiation dose of 64 Gray. The mean time between radiation and first presentation of AUF was 88 months [18–192 months]. The most frequent oncological diagnosis was bladder cancer in men (21%) and cervical cancer in women (18%). A medical history of (endo)vascular treatment was present in 33%. Indwelling ureteral stents were present in 29 patients (88%), reasons for placement were ureteral strictures due to radiation, retroperitoneal fibrosis or ureteral–ileal stenosis.

Hematuria was observed in all patients; 9% intermittent microhematuria (3/33 patients), and 91% (30/33 patients) building up to or initially presenting with massive hematuria. Accompanying symptoms were clot retention (21%), flank pain (9%), shock (6%), sepsis (3%), melena (3%), and urosepsis (3%).

All patients underwent a cumulative number of 40 investigations (mean 1.2 per patient) not leading to AUF diagnosis. For confirmation of the diagnosis of AUF, angiography was most decisive (19 times), followed by CT angiography (8 times), during surgery (4 times), ureterography (2 times), MRI (1 time), and postmortem (1 time).

Based on the details of clinical presentation, applied diagnostics and treatment, we presented a clinical algorithm, as presented in Supplementary Fig. 3.

Supplementary Fig. 4 shows the AUF locations. The common iliac artery was predominantly involved (73%), right sided in 38% and left sided in 35%. At diagnosis, seven patients with a medical history of vascular surgery on one side, such as thrombendarteriectomy or stenting, showed the AUF on the ipsilateral side of the repair and in 4 out of 7 patients a pseudoaneurysm was observed. In all six patients after central vascular repair, the AUF was found on the right side a pseudoaneurysm seen in three cases.

### *Treatment, follow-up, and outcomes (Table **2**)*

Open vascular repair was performed in 10/33 patients (30%), urological treatment in 7/33 patients (21%), and endovascular stent graft placement in 24/33 patients (73%) with embolization of the internal iliac artery in eight patients.

A combined urological and (endo)vascular treatment was performed in 12/33 patients (36%).

In two patients nephroureterectomy was performed, as hematuria was considered to be of renal origin. Afterwards, AUF appeared to be the cause of the hematuria.

Follow-up ranged from 1.5 days to 92 months with a median of 35 months. Fifteen patients died during follow-up; two were AUF related, five were sepsis related, and eight were due to other causes.

The first AUF-related death was a patient with Chronic Obstructive Pulmonary Disease (COPD) Gold IV, who died 6 days after placement of an endovascular stent, due to sepsis and weaning difficulties on the ICU. The second AUF-related death was due to severe hemorrhage which was inadequately controlled. This patient died within 2 days after presentation and the definitive diagnosis was found at autopsy.

The five sepsis-related deceased patients died after 1–4 months (mean 3 months). Their deaths were possibly related to the initiation of infection after insertion of endovascular stent grafts (3×) or vascular prosthesis during AUF treatment.

The causes of death not related to AUF were: metastatic disease in three patients after 11–34 months (mean 25 months), cardiovascular causes in two patients after, respectively, 35 and 50 months, a car accident in one patient after 84 months, probably COVID-19 in one patient after 41 months, and intestinal pneumatosis in one patient after 1 month.

This results in an overall mortality rate of 45% after a mean of 23 months, with a AUF-specific mortality rate of 6%.

In four patients, a second AUF developed after 1 day and 4, 8, 12 months, respectively. All four patients were treated with an endovascular stent graft and survived with a follow-up of 92, 32, 50, and 15 months, respectively. One patient developed an arterio-conduit fistula (ACF) after 6 months, which was treated with an endovascular stent, and had an uneventful follow-up of 30 months. Besides these five patients with second AUF or ACF development, none of the other patients showed persistent hematuria.

## Discussion

This nationwide cross-sectional questionnaire analysis comprehensively presents the occurrence of AUF in The Netherlands. Based on the results, risk factors, location, and clinical presentation of AUF was described. We also presented an overview of performed diagnostic modalities, and treatment of AUF in a modern healthcare system as in The Netherlands.

Our main finding is the incidence of AUF in The Netherlands per year. The calculated incidence in The Netherlands of AUF was 3.5 AUFs/year. The true incidence is likely to be higher, considering the non-response by 38% of urologists. In case the incidence of AUF in the practices of non-responders was comparable to those of the responders, incidence of AUF would actually be 5.6 per year (90/16). Calculations have to be interpreted with caution. The questionnaire was only sent to urologists. Some cases of AUF where urologists were not involved might have been missed. In addition patients may have died because of AUF in whom the diagnosis has not been made at all. Finally, recall bias may have played a roll.

In the literature, no figures of incidence changes over the past few years can be found. However, larger series have been published in recent years.

Krambeck et al. [4] only describe seven AUF patients between 1975 and 2004, while Omran et al. [5] and Simon et al. [6] describe 25 AUF patients between 2011 and 2020 and 16 AUF patients between 2005 and 2020, respectively. This suggests that the reported incidence is increasing.

This increase may be due to more extensive pelvic treatments for varying oncological and vascular procedures in current surgical medicine, increased treatment with ureteral indwelling stents, more frequent radiation therapies, and overall longer life expectancy. Other possible reasons are improving imaging modalities, and improvement of medical registration and electronic patient files, leading to easier identification of AUF cases. It is difficult to estimate the true incidence based on the numbers of published cases, because publication bias cannot be ruled out.

All our patients had a history of oncological treatment, irradiation therapy, vascular pelvic surgery, or chronic ureteral indwelling stents. Thus, all our patients had fistulas secondary to other conditions. This is in accordance with the findings described in Bergqvist and Madoff [7, 8], who found that 85% of the fistulas were secondary fistulas. Primary fistulas are probably rare or may remain unrecognized.

**Fig. 1:**
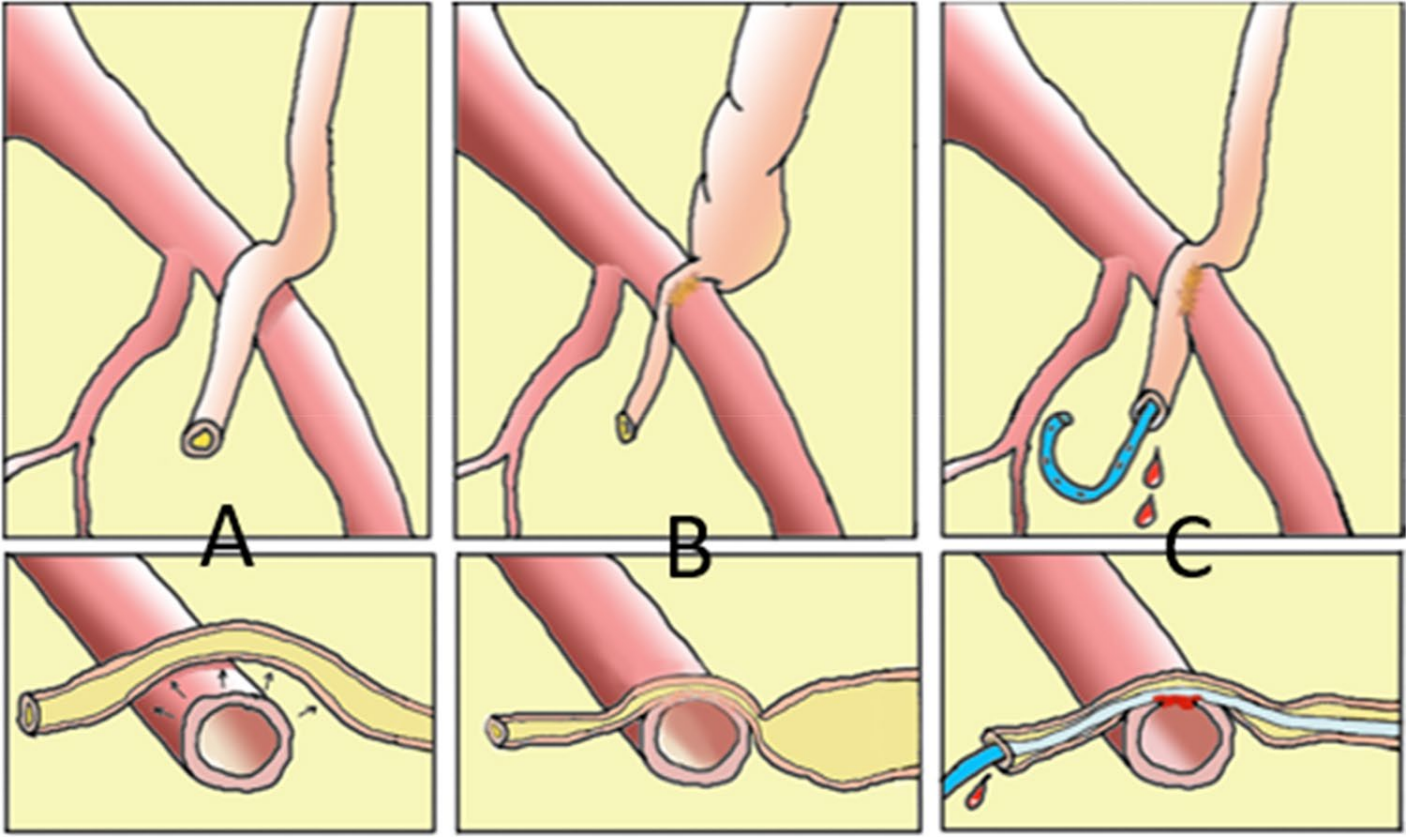
Illustration to explain the pathophysiology of AUF. **A** Normal condition: a freely movable ureter with a pulsatile artery. The artery not affecting the ureter. **B** Pelvic surgery and/or radiation could cause fibrosis and ischemic injury. This leads to ureter obstruction and hydronephrosis and fixation of the ureter to the arterial wall. **C** Ureteral stent placement to treat hydronephrosis which causes friction due to less freely movable ureter. In time, fibrosis, ischemia and/or friction could cause localized necrosis and eventually AUF

Although the pathophysiology of AUF is still uncertain, different hypotheses exist on the development of AUF, and the role of risk factors (Fig. 1). Surgery and radiation therapy lead to ischemia and fibrosis with fixation of a frail ureter to an artery (Fig. 1b). Ureteral indwelling stents can increase the damage due to pressure, facilitating necrosis and, eventually, fistula formation (Fig. 1c) [8, 9].

The male to female ratio was 55%:45%. This is in contrast with most cohort studies, such as Subiela et al. [10], who found a female predilection (59.6%). An explanation could be that bladder cancer was the predominant type of cancer and this is more common in the male population.

The clinical presentation in the majority of our patients was long-lasting intermittent (micro)hematuria that promptly shifted to massive hemorrhage, especially during ureteral stent extraction or replacement; only three cases (9%) had intermittent microhematuria without clinical consequences as the only symptom. This clinical presentation mirrors that of previous studies [11].

Several abdominal imaging modalities failed to confirm the diagnosis AUF. In our patient population cohort 38 investigations/imaging procedures were performed which not lead to the correct diagnosis. In two CT angiographies renal clots suggested a renal bleeding, leading to unnecessary nephroureterectomy. Angiography seems to be the most efficient modality, confirming diagnosis in 19/33 patients (58%). However, a negative angiography could not exclude AUF, because active bleeding is necessary for reliable angiography [12]. In case of negative angiography, provocative angiography (ureteral or vascular manipulation at AUF site) should be considered under controlled circumstances [12]. Diagnosis of AUF remains challenging because of low sensitivity of imaging tests.

Therapeutic management in The Netherlands is predominantly endovascular (73%) with the placement of stent grafts during angiography. In this century there has been a shift in the therapeutic management of AUF from a surgical toward a less invasive approach [9]. In the last decade, combination of therapies are more frequently performed. In the acute phase vascular and urologic surgeons work together in open surgery or the interventional radiologist starts with placing an endovascular stent graft and the vascular surgeon follows with a delayed open reconstruction (extra-anatomic bypass) or treating of graft infection and stent thrombosis [5, 13]. One of the measures to prevent future fistula formation may include considering nephrostomy instead of chronic indwelling stent in patients with multiple risk factors. A multidisciplinary approach is important for adequate diagnosis and treatment.

The AUF-specific mortality rate in our cohort was 6%, which is at the lower end of the spectrum. Five patients had sepsis-related death likely due to the endovascular stent grafts placed during AUF-treatment. However, this occurred after more than 30 days, so was not calculated in the AUF-specific mortality.

In previous studies the reported AUF-related mortality was rather variable: Escobar et al. found 10–13% and 22% in the hemodynamic instable patients [14], van den Bergh et al. described 13% AUF-related mortality [1], and more recently published Heers et al. calculated an AUF-related mortality of 7.7% [9].

The overall mortality was 45% after a median of 4 months. A possible explanation for this high rate is limited overall prognosis due to oncological and/or vascular comorbidity [15].

Noteworthy, in our cohort four new secondary AUFs were seen within 1 year and one ACF, all successfully treated by an endovascular stent. Simon et al. [6] and Omran et al. [5] describe recurrent AUFs, but no literature was found on new secondary AUFs. A possible explanation could be that these patients were > 70 years and all had radiation therapy in the past.

### Strengths and limitations

To our knowledge this is the first nationwide cross-sectional questionnaire analysis for AUF and the largest series of new AUF patients. One of the main strengths is that the response rate of 62% is quite high compared to overall response rate on similar questionnaires. This adequately reflects all urologists in The Netherlands.

Our study is the first to address the occurrence of AUF and contemporary experience by urologists on a national level. However, our study is limited by recall bias, possible missed diagnosis, and the retrospective nature.

## Conclusions

The estimated incidence of AUF in the time period 2003–2018 in The Netherlands is close to 4 AUFs per year, but the true rates may be higher. In 56 cases described in this series, hematuria, sometimes massive, was the most predominant initial symptom. Angiography appears the best diagnostic modality. Treatment was predominantly endovascular intervention.

We identified previous oncological and vascular treatment as well as indwelling ureteral catheters as major risk factors. Reported mortality rate was 6% and may be underestimated due to missed diagnoses.

AUF should be considered in patients with persistent intermittent or massive hematuria, especially in combination with history of oncologic (radiation) therapy, as well as vascular surgery.

Clinical awareness of AUF is important because of its possibly life threatening nature. A multidisciplinary approach is important in diagnosis and treatment.

## Supplementary Information

Below is the link to the electronic supplementary material.

Supplementary file1 (DOCX 97 KB)

Supplementary file2 (DOCX 47 KB)

Supplementary file3 (DOCX 49 KB)

Supplementary file4 (DOCX 81 KB)

## Abbreviations

ACF: Arterio-conduit fistula
AUF: Arterio-ureteral fistula
CT: Computed tomography
