# Supplementary figures and images for: Arterio-ureteral fistula: a nationwide cross-sectional questionnaire analysis
Source: World J Urol. 2022 Jan 22;40(3):831–9. doi: 10.1007/s00345-021-03910-3 (PMC8783176; doi:10.1007/s00345-021-03910-3)

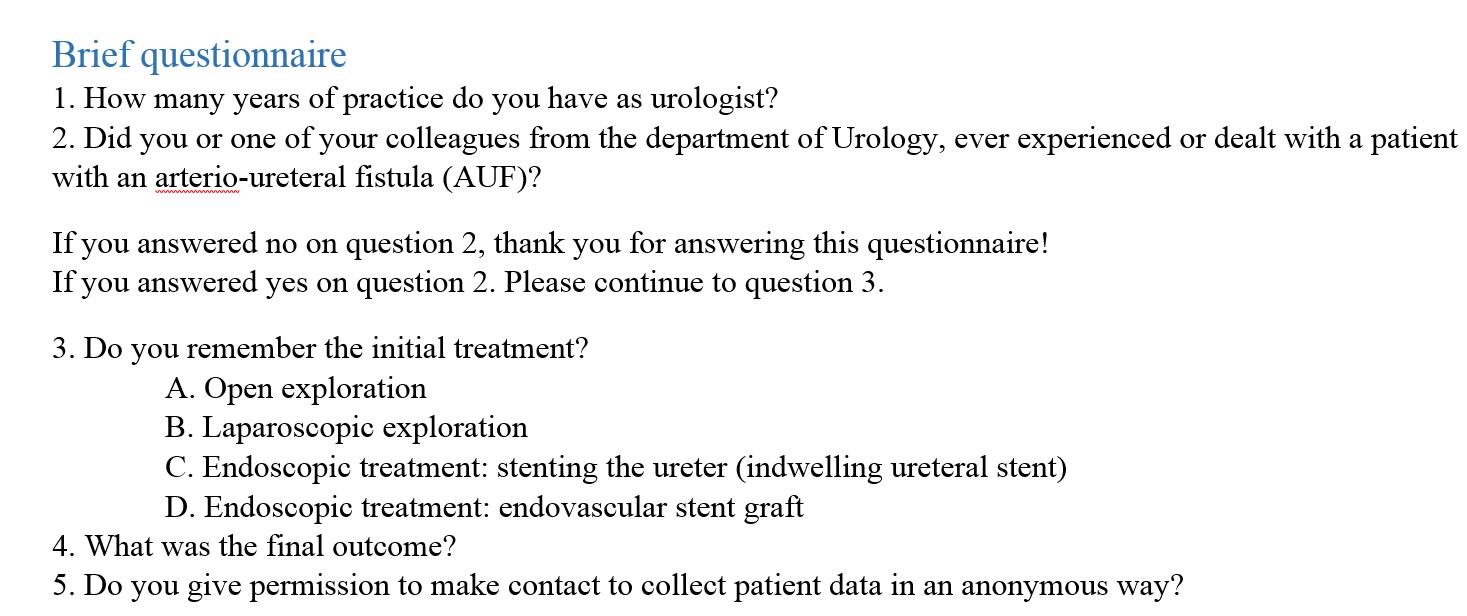


**Figure 1: brief questionnaire sent to all Dutch urologists**

Supplement: Supplementary file 1 — Supplementary file1 (DOCX 97 KB) [file 345_2021_3910_MOESM1_ESM.docx]

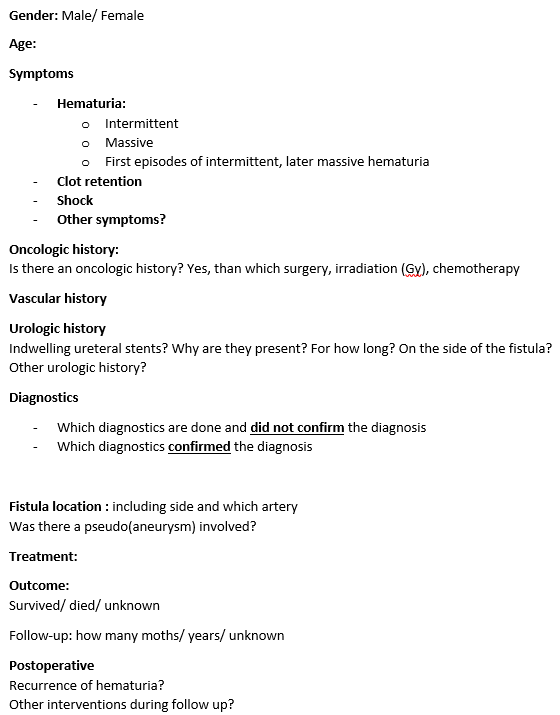


Supplementary Figure 2: Standard item list

Supplement: Supplementary file 2 — Supplementary file2 (DOCX 47 KB) [file 345_2021_3910_MOESM2_ESM.docx]

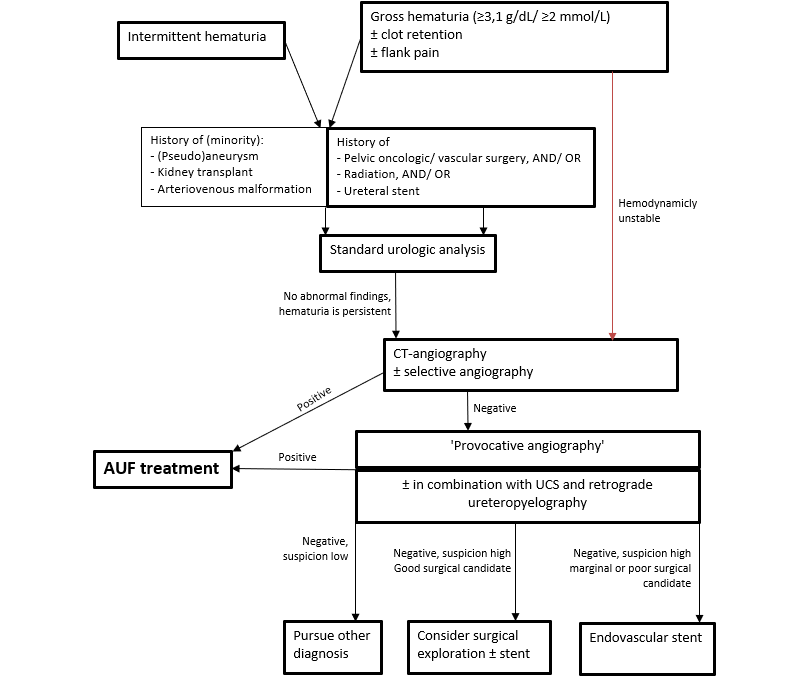


**Figure 3: Diagnostic algorithm**

Supplement: Supplementary file 3 — Supplementary file3 (DOCX 49 KB) [file 345_2021_3910_MOESM3_ESM.docx]
