# Supplementary material for: Arterio-ureteral fistula: a nationwide cross-sectional questionnaire analysis
Source: World J Urol. 2022 Jan 22;40(3):831–9. doi: 10.1007/s00345-021-03910-3 (PMC8783176; doi:10.1007/s00345-021-03910-3)
Supplement: Supplementary file 4 — Supplementary file4 (DOCX 81 KB) [file 345_2021_3910_MOESM4_ESM.docx]

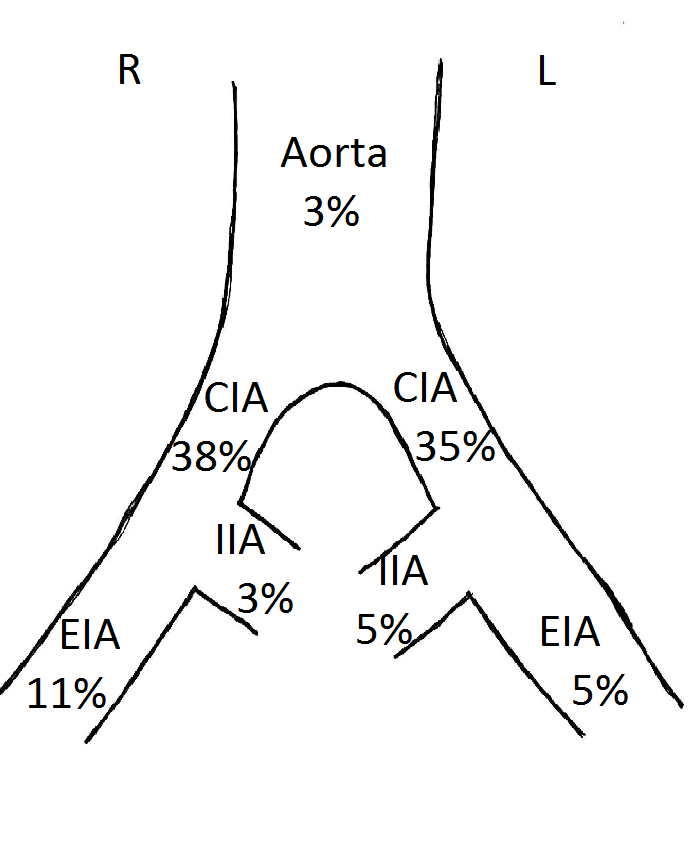


**Figure 4: location of AUF. CIA: common iliac artery, EIA: external iliac artery, IIA: internal iliac artery, L: left, R: right**
